# Abrupt Bølling warming and ice saddle collapse contributions to the Meltwater Pulse 1a rapid sea level rise

**DOI:** 10.1002/2016GL070356

**Published:** 2016-09-14

**Authors:** Lauren J. Gregoire, Bette Otto‐Bliesner, Paul J. Valdes, Ruza Ivanovic

**Affiliations:** ^1^ School of Earth and EnvironmentUniversity of LeedsLeedsUK; ^2^ School of Geographical SciencesNCARBoulderColoradoUSA; ^3^ University of BristolBristolUK

**Keywords:** sea level rise, North American ice sheet, abrupt warming, Meltwater Pulse 1a, Bølling

## Abstract

Elucidating the source(s) of Meltwater Pulse 1a, the largest rapid sea level rise caused by ice melt (14–18 m in less than 340 years, 14,600 years ago), is important for understanding mechanisms of rapid ice melt and the links with abrupt climate change. Here we quantify how much and by what mechanisms the North American ice sheet could have contributed to Meltwater Pulse 1a, by driving an ice sheet model with two transient climate simulations of the last 21,000 years. Ice sheet perturbed physics ensembles were run to account for model uncertainties, constraining ice extent and volume with reconstructions of 21,000 years ago to present. We determine that the North American ice sheet produced 3–4 m global mean sea level rise in 340 years due to the abrupt Bølling warming, but this response is amplified to 5–6 m when it triggers the ice sheet saddle collapse.

## Key Points

- The cause of the largest rapid sea level rise ever recorded, Meltwater Pulse 1a (14.6 ka), is unresolved
- We quantify how much and by what mechanisms the North American ice sheet could have contributed to Meltwater Pulse 1a
- The Bolling warming likely triggered the saddle collapse mechanism contributing 5‐6 m or more to MWP1a from the North American ice sheet

## 1 Introduction

Since the Last Glacial Maximum (LGM) around 23–21 thousand years ago (ka), the Earth underwent a major transition into the current interglacial period, during which the North American and Eurasian continents deglaciated entirely, and the Greenland and Antarctic ice sheets, as well as glaciers worldwide, retreated. In total, this produced around 130 m of global mean sea level rise (GMSLR) [*Lambeck et al*., 2014], which was sometimes contributed to by major episodes of accelerated ice melt. Meltwater Pulse 1a (MWP1a) is the largest of these, identified as a 14–18 m of GMSLR in less than 340 years at 14.6 ka [*Deschamps et al*., 2012] in coral reef records from Tahiti and Barbados, as well as other sea level proxies around the world. This event also occurred around the time of an abrupt Northern Hemisphere warming of 4–5°C that took place within a few decades to centuries [*Buizert et al*., 2014; *Deschamps et al*., 2012]. However, the link between this intense ice melt and warming remains elusive. This is partly because of the imprecise chronology of events, and also because the origin of the MWP1a is uncertain. Ice melt can significantly disturb ocean circulation producing widespread changes in surface climate, but this impact is different (and can be opposite) depending on whether the ice melt goes into the Arctic, North Atlantic, or Southern Ocean [*Clark*, 2001; *Ivanovic et al*., 2016; *Menviel et al*., 2011; *Peltier et al*., 2006]. It is therefore important to know how much the respective ice sheets each contributed to the event.

The source of MWP1a has previously been assessed through fingerprinting the pattern of sea level rise/fall caused by the change in gravitational pull exerted by an ice mass on oceans. Although initial studies rejected a major North American ice sheet (NAIS) source, recent work overturned this finding and suggested it could actually have contributed up to 10 m to the total sea level change in 340 years [*Gomez et al*., 2015]. However, the large uncertainties in sea level reconstructions make it impossible to discriminate between a major NAIS contribution and a 100% contribution from the Antarctic ice sheet [*Gomez et al*., 2015; *Liu et al*., 2016].

For the Antarctic ice sheet, direct constraints on changes in thickness or extent around the time of MWP1a are limited and debated. Southern Ocean records of iceberg‐rafted debris [*Weber et al*., 2014] show that the largest iceberg fluxes occur around the time of MWP1a (14.6 ka). Moreover, numerical ice sheet modeling [*Golledge et al*., 2014] suggests that Southern Ocean overturning triggered up to 2 m sea level equivalent of ice loss in Antarctica in 340 years, a significant but relatively small contribution to the 14 m GMSLR at MWP1a [*Deschamps et al*., 2012]. In contrast, North American ice sheet reconstructions show major ice sheet changes around the time of MWP1a [*Dyke*, 2004; *Gowan et al*., 2016; *Peltier et al*., 2015; *Tarasov et al*., 2012]. Moreover, *Gregoire et al*. [2012, hereafter “G12”] provided a mechanistic explanation for a major NAIS contribution to this event, showing that the Cordilleran‐Laurentide ice sheet separation caused accelerated ice melt due to a height‐mass balance feedback triggered by gradual climate forcing. This “saddle collapse” mechanism in North America produced 7 m of GMSLR in 350 years (10 m in 500 years).

From geological constraints, exactly when the separation of the two ice sheets took place is uncertain [*Dyke*, 2004]. It is clear from the synthesis of these data that the separation of the Cordilleran and Laurentide ice sheet, which caused the saddle collapse and accelerated ice melt, could not have occurred after 14 ka and thus could not have corresponded to Meltwater Pulse 1b (11.3 ka) [*Abdul et al*., 2016]. The separation of the two ice sheets likely occurred between 16 ka and 14 ka [*Dyke*, 2004], overlapping with the timing of MWP1a (~14.6 ka). Thus, G12 suggested that the Cordilleran‐Laurentide saddle collapse could have contributed around half of MWP1a.

Given their close timing, it is compelling to think that at least part of MWP1a could also have been linked to the abrupt Bølling warming in the Northern Hemisphere, through accelerated ice melt in North America and Europe. *Carlson et al*. [2012] suggested that this abrupt warming would have caused a total of 6.9 m GMSLR in 500 years in North America, but their result did not account for dynamical or elevation‐melt feedbacks, which are likely to have intensified the melt rates. In short, it remains unclear how the Bølling warming and the saddle collapse are related.

Here we provide a mechanistically based statistical assessment of the possible range of North American ice sheet contribution to MWP1a from both the saddle collapse mechanism and the Bølling warming.

## 2 Methods

We ran ensembles of model experiments simulating North American ice sheet evolution over the last deglaciation (21–7 ka), using the Glimmer‐CISM 3‐D thermodynamic ice sheet model version 1.14 [*Rutt et al*., 2009]. This setup is based on G12, except that we vary ice model parameters and use different climate forcing methods and input data. The model uses the shallow ice approximation, which limits its ability to simulate processes at the marine margin or narrow ice streams. This approximation makes the model fast, allowing ensembles of several hundreds of experiments to be run over a full glacial‐interglacial cycle (120–0 ka). The ice sheet mass balance is computed with a positive degree day mass balance scheme using monthly mean temperature and precipitations from general circulation model (GCM) output spanning the period. The potential impact of proglacial lakes on meltwater discharge and ice dynamics is not simulated in this model.

### 2.1 Climate Forcing

**Figure 1:**
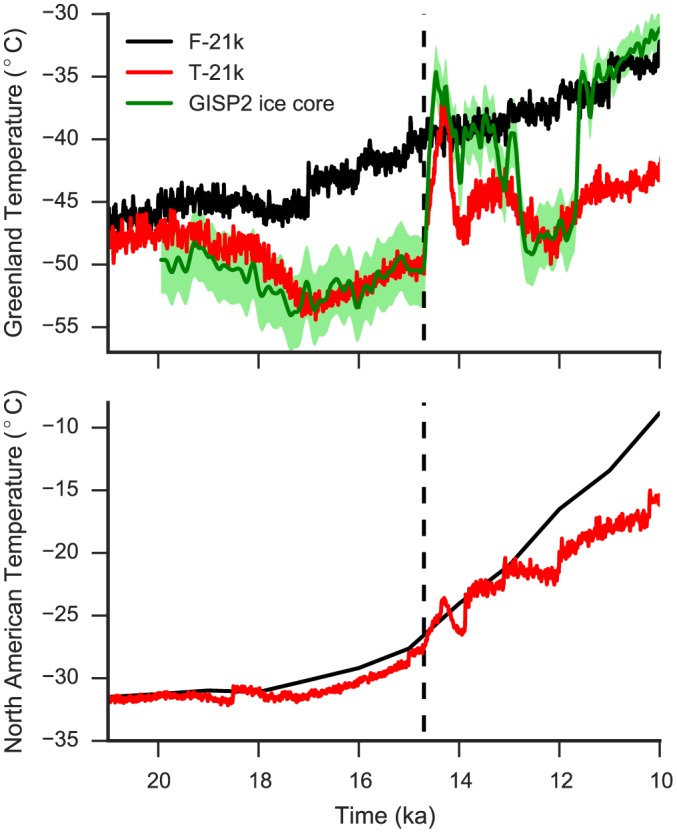
(top) Greenland and (bottom) North American temperatures in the F‐21k experiment (black), T‐21k experiment (red; CCSM3), and the GISP2 temperature reconstruction (green) [*Buizert et al*., 2014]. The T‐21k Greenland temperature is shifted by −9°C to reflect elevation difference between the T‐21k simulation and GISP2. The onset of the Bølling warming in the T‐21k experiment (14.7 ka) is shown by the dashed line.

We forced the ice sheet model with transient simulations of the last 21 thousand years run with two different GCMs, FAst Met Office/UK Universities Simulator (FAMOUS) and Community Climate System Model 3 (CCSM3), allowing us to account for some degree of climate uncertainty. The climate experiments were forced with similar boundary conditions for changes in greenhouse gases, orbit, and ice sheets (see respective references below). However, they used different scenarios for how much, where, and when the melting from ice sheets freshened the ocean. As a result, the climate evolves differently in the two simulations:

1. The trace‐21 ka experiment run with CCSM3 (henceforth “T‐21k”) simulates a rapid warming similar in magnitude and spatial pattern to the Bølling warming event [*Liu et al*., 2009, 2012] (Figure 1).
2. The FAMOUS transient experiment as used in G12 (henceforth “F‐21k”) reproduces well the overall slow climatic change during the deglaciation but does not simulate millennial scale climate change, including the Bølling warming event [*Gregoire et al*., 2012] (Figure 1).

In both climate experiments, sudden and artificial surface temperature changes occur when and where the ice sheet extent is updated in the models, due to surface albedo shifts. This ice mask (and bathymetry, land mask and surface elevation) is updated every 1000 years in the FAMOUS experiment and at irregular intervals in the Trace‐21k experiment. As in G12, we process the FAMOUS climatic fields to remove these stepped climate artifacts before they are input to Glimmer. This is done by averaging each monthly field over 1000 year intervals and interpolating linearly between these averages. We did not process the Trace‐21k data in the same way since we are specifically interested in the effect of the real abrupt climate changes in these experiments. Instead, we take the conservative approach of disregarding any abrupt change in ice sheet mass balance that occurs soon after a sudden change in the Trace‐21k ice mask.

For the F‐21k forcing, we drive the Glimmer‐CISM ice sheet model directly using model output climate fields (absolute forcing), as in G12 (*Fabs* forcing). For the T‐21k transient climate, we adopt two forcing methodologies (see section 2): (i) an absolute forcing as in G12, where we directly use the GCM's monthly temperature and precipitation fields (*Cabs* forcing) and (ii) an anomaly forcing, where we correct for present‐day model biases in the GCM's climatology (*Cano* forcing).

The anomaly forcing provides the means to correct for overbuilding of ice due to cold biases in the T‐21k experiment occurring under present‐day conditions. Such high‐latitude temperature biases are smaller in the F‐21k forcing, limiting the need for bias corrections. In addition, earlier tests show that using anomaly forcing with this model produces significantly worse results [*Gregoire*, 2010]. Therefore, we do not adopt the anomaly forcing with F‐21k in this analysis. All of our experiments start with a spin‐up phase corresponding to ice growth over the glacial cycle from 120 ka to 21 ka. The full forcing methodology is described in Text S1 in the supporting information.

### 2.2 Ensemble, Sampling Method, and Selection of Good Models

Uncertainties in ice sheet modeling are accounted for by running ensembles of ice sheet experiments, systematically varying model parameters within their feasible ranges following the method of *Gregoire et al*. [2011]. We sample the parameter values using a maximin Latin Hypercube Sampling technique [*Mckay*, 1992]. For this, ice mass balance and dynamic model parameters (Table S1) that have previously been shown to have the greatest influence on ice sheet geometry and evolution were selected [*Gregoire*, 2010]. Parameter values were sampled within uniform distributions with ranges of values (Table S1) identified in previous work [e.g., *Gregoire*, 2010].

We select ensemble members based on their LGM ice volumes and their evolving extent throughout the deglaciation [*Dyke*, 2004] (Figure S1). Reconstructed North American ice sheet volume ranges from 23 × 10^6^ km^3^ to 35 × 10^6^ km^3^ at the LGM, equivalent to 60–88 m GMSLR [*Clark and Tarasov*, 2014; *Lambeck et al*., 2014; *Peltier et al*., 2015; *Tarasov et al*., 2012]. However, reconciling constraints on individual ice sheet volume with constraints on global sea level change is difficult (the so‐called “missing ice problem”) [*Clark and Tarasov*, 2014]. Geologically constrained reconstructions suggest that the Antarctic ice sheet was responsible for 5–15 m GMSLR since the LGM [*Peltier et al*., 2015; *Tarasov et al*., 2012; *Whitehouse et al*., 2012; *Clark and Tarasov*, 2014]. However, this is at least 8 m lower than the Antarctic GMSLR contribution (23 m) estimated by *Lambeck et al*. [2014]. We therefore consider the possibility that the North American ice sheet is underestimated by up to 8 m in current reconstructions. Based on this we discard simulations with volumes lower than 23 × 10^6^ km^3^ and higher than 38 × 10^6^ km^3^ at the LGM (60–96 m GMSLR).

We compare the ice extent through the deglaciation with the reconstruction from *Dyke* [2004] using a bespoke metric which accounts for dating uncertainties (Text S2). We limit the ice extent comparison to the period up to 13 ka, which encompasses the Bølling warming, because the T‐21k simulation does not reproduce the climate recovery at the end of the Younger Dryas (12 ka). Instead, cold Northern Hemisphere climate continues for several thousands of years (Figure 1), thus delaying ice sheet retreat in the Cabs and Cano ensembles after 12 ka. We consider that this bias would not affect the meltwater pulse caused by the Bølling warming at 14.6 ka.

**Figure 2:**
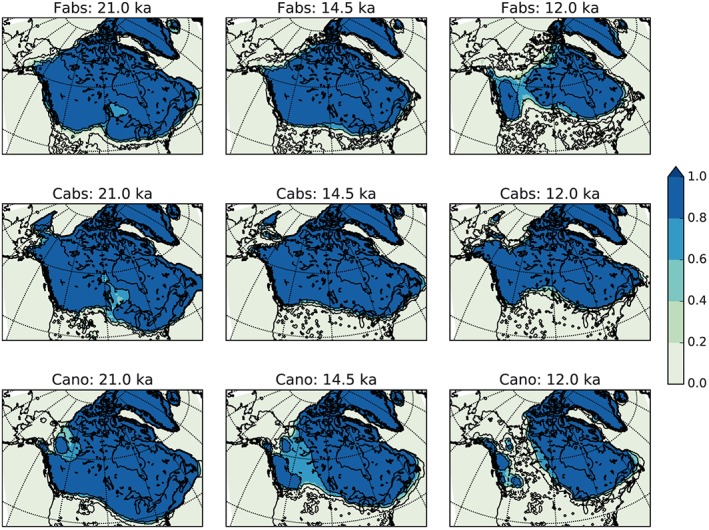
Ice extent averaged over the Not Ruled Out Yet ensemble members for the Fabs (FAMOUS absolute), Cabs (CCSM3 absolute), and Cano (CCSM3 anomaly) ensembles at 21, 14.5, and 12 ka. A fraction of 1 indicates areas where all ensemble members have ice, 0 where none have ice.

We defined a maximum acceptable extent error, to account for local or transient ice sheet features that are not taken into consideration by our model. In particular, the shallow ice approximation we use limits our ability to simulate the southeast Laurentide ice lobes at the LGM, our simple representation of calving tends to overestimate the marine ice extents, and the low resolution of the climate forcing (~500 km) tends to smooth out the details and local features in the reconstructed ice extent. We consider that such errors in our simulations are acceptable and set the maximum acceptable extent error through an iterative process by evaluating ice extent maps of the selected simulations. We discarded simulations with a cumulative extent error of more than 23% corresponding to 225,500 cells over 13 reconstructed maps (40.8 × 10^6^ km^2^ × century). The selected simulations have reasonable ice extents at the LGM (Figure 2) and through the deglaciation.

## 3 Results

### 3.1 Constraining the Deglaciation

**Figure 3:**
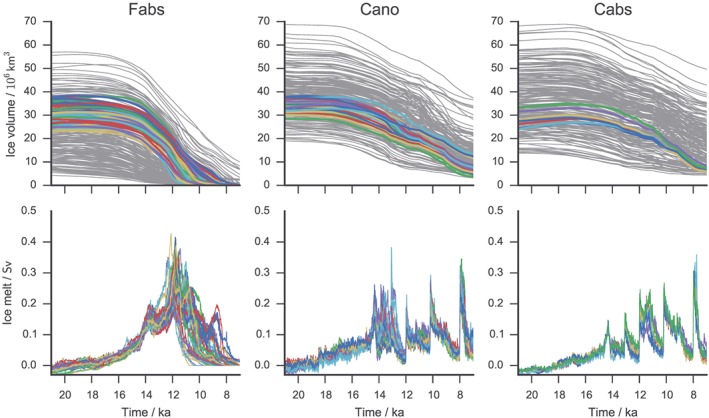
Evolution of ice volume and meltwater flux from the Not Ruled Out Yet ensemble members (in color) for each forcing through the deglaciation. The ice volume of all ensemble members is shown in grey in the top panels.

For each climate forcing (Fabs, Cabs, and Cano), we ran a perturbed physics parameter ensemble of 201 experiments, 603 simulations in total. After spin‐up, at 21 ka, we obtain ice volumes ranging from 0 to 70 × 10^6^ km^3^ (Figure S1). Moreover, we found that the rates of deglacial retreat depend on the forcing methodology (Figures 2 and 3) and the specific values of dynamical and mass balance parameters used.

To select the most realistic simulations, we apply constraints on ice extent evolution from the reconstruction of *Dyke* [2004] and on the North American ice sheet volume at the LGM as described in section 2. From the Fabs, Cano, and Cabs ensembles, 53, 25, and 8 simulations were selected, respectively (Figure S1). We refer to them as the Not Ruled Out Yet (NROY) ensemble of simulations following history‐matching terminology. The selected simulations have reasonable ice extents at the LGM and through the deglaciation (Figure 2).

Ice extent and thickness is similar between the Fabs and Cabs ensembles, as reflected in the NROY ensemble mean ice extent and thickness (Figures 2 and S2). The Cabs forcing, however, produces a slower rate of deglaciation (Figures 2, 3, and S2) than both Fabs and Cano forcing. Although the Cano forcing produces excess ice extent south of the Great Lakes at 21 ka, the ice extent evolution better matches the reconstruction of *Dyke* [2004] than the Fabs and Cabs forcings. In particular, the opening of the corridor between the Laurentide and Cordilleran ice sheets occurs prior to 14.0 ka in almost half of the NROY Cano experiments, consistent with ice extent reconstructions [e.g., *Dyke*, 2004], whereas this occurs 2–4 ka later in the Fabs and Cabs NROY ensembles. However, the ice surface elevation in the Fabs and Cabs ensembles is in better agreement with recent ice sheet reconstructions [*Peltier et al*., 2015; *Tarasov et al*., 2012]. Therefore, each forcing methodology has its strength and weakness in the ice evolution it produces, and we consider their results as all equally likely. Using these ensembles, we assess the plausible characteristics (amplitude, volume, duration, and timing) of meltwater pulses caused by the saddle collapse and by the abrupt Bølling warming.

### 3.2 Effect of the Saddle Collapse

**Figure 4:**
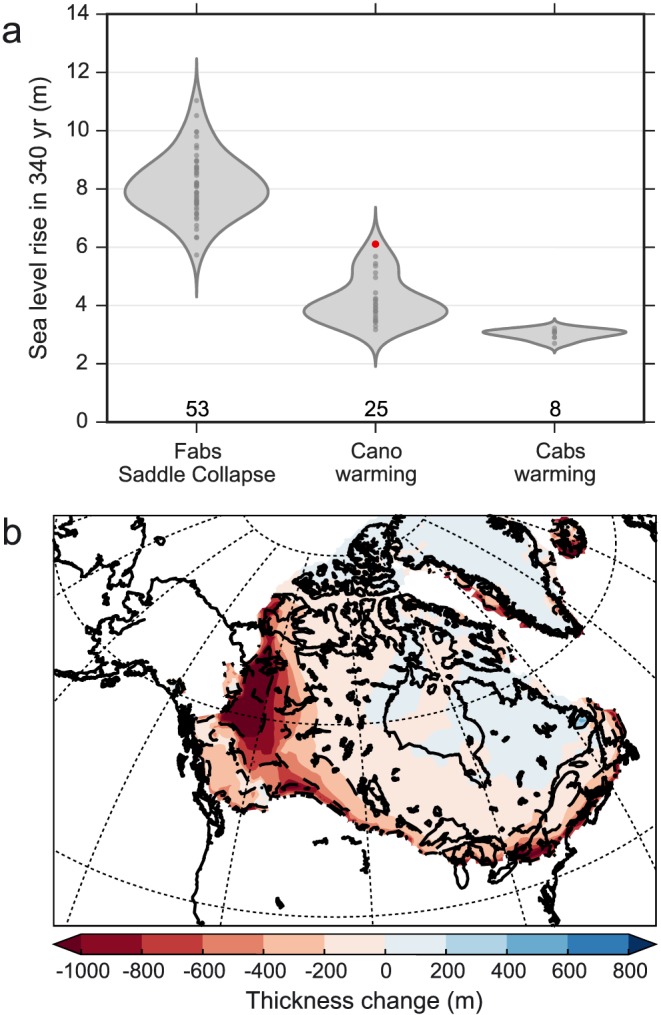
(a) Distribution of sea level rise in 340 years in Not Ruled Out Yet experiments (with number in each ensemble indicated in bold) resulting from the saddle collapse in the Fabs ensemble and from the abrupt Bølling warming in the Cano and Cabs experiments. (b) Ice sheet thickness change between 14.54 and 14.20 ka in the Cano experiment 116 (red dot in Figure 4a) when the Bølling warming triggers the saddle collapse.

The saddle collapse event occurs spontaneously during the North American deglaciation, when the Laurentide and Cordilleran ice sheets separate. To identify these events systematically, we detect the timing and amplitude of the largest peak in melt rate over the duration of each individual simulation. We then verified that this peak occurred during the separation of the Cordilleran and Laurentide ice sheet in the experiments by plotting the location of the thickness changes during the event. Finally, we calculated the GMSLR from ice volume change in the 340 years period centered around the time of the maximum melt rate (Figure 4), assuming an ocean area of 360,768,600 km^2^.

The T‐21k forcings have artificial warming steps caused by GCM boundary condition changes being implemented at irregular intervals, which interfere with the timing and amplitude of the saddle collapse event. We were therefore only able to perform this analysis on the Fabs ensemble (not the Cano and Cabs ensembles), as its forcing was smoothed to remove such artifacts (see G12 methods).

The timing of the saddle collapse meltwater pulse varies within the ensembles of Fabs NROY experiments but always occurs between 12.3 ka and 10.9 ka, with a maximum meltwater flux of 0.25 to 0.43 Sv producing 5.7 to 11.0 m GMSLR in 340 years. The exact timing and amplitude of the pulse is a result of many factors, and we have not found a clear relationship with ice sheet geometry or any specific parameter values.

### 3.3 Effect of the Abrupt Bølling Warming

The abrupt Bølling warming in the T‐21k forcing starts at 14.7 ka with maximum North American temperature at 14.3 ka. Following this, there is an acceleration in melt rate in the NROY Cano and Cabs ensembles (Figure 3), with consistent timing of the maximum melt rate around 14.3 ka, coinciding with the peak in warming over North America. We calculate the maximum GMSLR in a 340 year moving window between 14.7 and 13.85 ka to account for variable durations in the meltwater pulses and allow for a direct comparison with sea level records.

The GMSLR produced is 3.2 to 6.1 m with the Cano anomaly forcing and 2.7–3.2 m with the Cabs absolute forcing. The amplitude of sea level rise partly depends on the positive degree day factor for ice, which directly affects the amount of melt per °C warming, and the lapse rate, which influences the areal extent of ablation. The shape of the ice sheet immediately before the warming, which is dependent on model parameters and forcing methodology, also impacts the amplitude of the pulse through its control on ablation area.

In five of the Cano simulations, the separation of the two ice sheets occurs less than 500 years after the end of the warming (between 14.0 and 13.8 ka). These are the simulations that produce the cluster of high‐end GMSLR (5.1 to 6.1 m) in the Cano ensemble (Figure 4a). This is because the abrupt warming starts melting a large proportion of the saddle between the Cordilleran and Laurentide (Figure 4b) and triggers the saddle collapse. In the remaining Cano experiments, the two ice sheets are either already fully separated at the time of the Bølling warming, or the saddle between the Cordilleran and Laurentide ice sheets remains too thick to be destabilized by the abrupt warming (also the case for all the Cabs experiments), and only 3.0 to 4.4 m GMSLR is produced in either case. In general, the smaller the warming meltwater pulse is, the later the saddle collapse occurs.

The best ice extent scores in the Cano ensemble are obtained when the Cordilleran‐Laurentide ice free corridor opens close to 14 ka. Those are the experiments that produce the largest response to the abrupt Bølling warming. Simulations with a pre‐15 ka separation have the smallest LGM volumes and extent, and larger misfits to the ice extent reconstruction (extent errors) than other NROY experiments.

In the simulations with an early (pre‐Bølling warming) separation of the Cordilleran and Laurentide ice sheets, the melt associated with the abrupt warming is located predominantly in the ice free corridor. Otherwise, the melt is mainly on the northern part of the saddle linking the two ice sheets. In both cases, this melt would be routed through the Mackenzie River toward the Arctic Ocean [*Wickert*, 2016]. Melt also occurs southeast of the Laurentide ice sheet in our experiments and would be routed through the Hudson and Mississippi Rivers toward the North Atlantic [*Wickert*, 2016]. Proglacial lakes likely stored a small part of the ice sheet melt (less than 0.06 m of sea level equivalent) [*Teller et al*., 2002].

The F‐21k (Fabs) forcing does not include a Bølling warming signal and so cannot be used to assess the climate event's influence on North American ice sheet evolution.

## 4 Discussion and Conclusion

Our results suggest that the North American ice sheet could have contributed to MWP1a through two different mechanisms: the ice saddle collapse caused by the separation of the Cordilleran and Laurentide ice sheets (described in G12), which we associate with the timing of MWP1a, and accelerated melt from the abrupt Bølling warming in the Northern Hemisphere at 14.6 ka. The separation of the two ice sheets on its own can produce a meltwater pulse of 5.7–11.0 m GMSLR in 340 years associated with the saddle collapse. However, in these experiments, the separation of the two ice sheets occurs after 12 ka, whereas geological reconstructions indicate the separation occurred between 16 and 14 ka [*Dyke*, 2004; *Gregoire et al*., 2012]. This delay is probably due to biases or missing processes in the climate and ice sheet models (e.g., the use of shallow ice approximation and simplifications in the mass balance modeling) as discussed in *Gregoire et al*. [2015]. The separation of the Cordilleran and Laurentide ice sheets thus occurred either before, during, or shortly after the Bølling warming (14.6 ka).

When the Cordilleran and Laurentide ice sheets are already separated at the time of the Bølling, the abrupt warming produces 3–4 m GMSLR in 340 years. This response is amplified by 2/3 (5–6 m) when the Bølling warming triggers the separation (the so‐called saddle collapse mechanism; G12). These simulations produce the best match to geological data. Thus, these results strongly suggest that the Bølling warming triggered the saddle collapse mechanism contributing 5–6 m or more to MWP1a from the North American ice sheet. Additional processes not included in this study, such as marine ice sheet instability, interaction with proglacial lakes, and “higher‐order” ice stream dynamics, could have enhanced ice loss leading to a potentially larger North American ice sheet contribution to MWP1a.

Constraining the timing of the separation of the Cordilleran and Laurentide ice sheets with respect to the Bølling warming is crucial to narrowing down the potential North American contribution to MWP1a and to confirm the mechanisms that caused the most rapid sea level rise in our geological past.

## Supporting information



Supporting Information S1
