# Supplementary material for: Abrupt Bølling warming and ice saddle collapse contributions to the Meltwater Pulse 1a rapid sea level rise
Source: Geophys Res Lett. 2016 Sep 14;43(17):9130–7. doi: 10.1002/2016GL070356 (PMC5053285; doi:10.1002/2016GL070356)
Supplement: Supplementary file 1 — Supporting Information S1 [file GRL-43-9130-s001.docx]

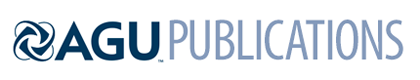


*Geophysical Research Letters*

Supporting Information for

**Abrupt Bølling warming and ice saddle collapse contributions to the Meltwater Pulse 1a rapid sea level rise**

L J Gregoire^1^, Bette Otto-Bliesner^2^, Paul J Valdes^3^ , Ruza Ivanovic^1^

1 University of Leeds, UK; 2 NCAR, Boulder, Colorado, USA; 3 University of Bristol

**Contents of this file**

Text S1 to S2

Figures S1 to S2

Tables S1 to S1

**Introduction**

This supplementary material includes additional methodological information (Text S1 and S2) as well as supplementary figures and tables.

## Text S1. Forcing methodology

All of our experiments start with a spin-up phase corresponding to ice growth over the glacial cycle from 120 ka to 21 ka. To emulate the climate change over this period, we interpolate between pre-industrial and LGM climate with an index representing glacial climate evolution. The index is built from the Greenland ice core temperature reconstruction of Kindler et al. (2014) as follows:

$$I(t) =\frac{T\left( t \right)-T\left( Holocene \right)}{T\left( 21 ka \right)- T\left( Holocene \right)}$$

Where T(Holocene) stands for the NGRIP Holocene values (241.6 K ; NGRIP members, 2004) as defined in the Kindler et al. (2014) temperature reconstruction.

# S1.1 Absolute forcing (Cabs and Fabs)

The spin-up climate forcing for absolute forcing is calculated in the following way:

$$T_{f}\left( x,y,m,t \right)= T_{clim}(x,y,m) + I\left( t \right)\text{×} \left( \left( T_{LGM}(x,y,m) + \gamma H_{LGM}(x,y,m) \right)- \left( T_{clim}(x,y,m) + \gamma H_{clim}(x,y,m) \right) \right)$$

$$P_{f}\left( x,y,m,t \right)=P_{clim}(x,y,m) \times\left( I\left( t \right)\times\left( \frac{P_{LGM}(x,y,m)}{P_{clim}(x,y,m)}-1 \right)+1 \right)$$

where *T* is surface temperature (°C); *P* is precipitation (mm day^-1^); *H* is orographic elevation in the model (metres) at the LGM (*LGM*) or corresponding to the reanalysis (*clim*); $\gamma$ is the lapse rate (-5 °C /km); *x* and *y* represent the latitude/longitude spatial dimensions (metres), *m* is an index for the months of the year (1-12; Jan-Dec) and *t* is the time (years).

This follows a standard method for parameterising climate change over glacial-interglacial cycles(Marshall et al., 2002; Zweck and Huybrechts, 2005). It accounts for the effect on temperature of differences in topography between the climate model, the reanalysis and the ice sheet model, through a lapse rate correction. In this absolute forcing, we do not correct the LGM climate input for any bias. However, for the spin-up phase of the experiments, instead of using the modelled pre-industrial climate in our interpolation, we use reanalysis data from present day (Environmental Modeling Center, 2010). This avoids early growth of the ice sheet at the last interglacial due to cold biases in simulated present day climate, but the climate forcing at 21 ka remains the absolute modelled climate at the LGM.

We start the deglacial simulations from the spin-up state at the Last Glacial Maximum and force the ice sheet model with the transient monthly *T* and *P* from the deglacial runs (Trace-21ka and FAMOUS). *Fabs* simulations start at 21 ka, which is the start of the FAMOUS experiment, and *Cabs* simulations start at 22 ka, which is the start of the Trace-21ka experiment.

# S1.2 Anomaly forcing (Cano)

The anomaly forcing is similar to the absolute forcing, except that the LGM climate state is corrected for the present day anomaly climate compared to the reanalysis. This is only applied to the Trace-21ka CESM climate data (to produce *Cano*) and not the FAMOUS climate output, which already simulates a reasonable LGM surface climate over North America and would not be improved by the bias correction.

Using the anomaly method, the spin-up forcing can be expressed as:

$$T_{f}\left( x,y,m,t \right)= T_{clim}(x,y,m) + I\left( t \right)\text{×} \left( \left( T_{LGM}(x,y,m) + \gamma H_{LGM}(x,y,m) \right)- \left( T_{PI}(x,y,m) + \gamma H_{PI}(x,y,m) \right) \right)$$

$$P_{f}\left( x,y,m,t \right)=P_{clim}(x,y,m) \times\left( I\left( t \right)\times\left( \frac{P_{LGM}(x,y,m)}{P_{PI}(x,y,m)}-1 \right)+1 \right)$$

The deglacial forcing for *Cano* is calculated in this way:

$$T_{ano}\left( x,y,t \right)= T\left( x,y,t \right)- \left( \left( T_{PD}\left( x,y,m(t) \right)+ \gamma H_{PD}\left( x,y,m(t) \right) \right)- \left( T_{clim}\left( x,y,m(t) \right)+ \gamma H_{clim}\left( x,y,m(t) \right) \right) \right)$$

$$P_{ano}\left( x,y,m,t \right)=P(x,y,m) \times\frac{P_{PD}(x,y,m(t))}{P_{clim}(x,y,m(t))}$$

As with *Cabs* simulations, *Cano* simulations start at 22 ka; the start of the Trace-21ka experiment.

# Text S2. Ice extent metric

We compare the ice extent through the deglaciation with the reconstruction from Dyke (2004), which provides isochrones of ice extent at specific radiocarbon dates during the deglaciation. Using the intcal program (Reimer et al., 2009), we define a range of calendar dates (*Tmin*, *Tmax*) that match the radiocarbon ages (*T*) from each slice assuming ±500 years uncertainty on radiocarbon ages of the isochrones following Tarasov et al. (2012). Having projected and regridded the data presented by Dyke (2004) onto the Glimmer North American grid, we compare binary ice extent fields (presence versus absence of ice) point by point with the following metric:

$$error = \sum_{T} \sum_{x} \sum_{y} {min}_{t}(|m\left( x,y,t \right)-r(x,y,T)|)$$

Where *m* is the model and *r* the reconstructed ice extent, which are equal to 1 where ice is present and 0 otherwise, and *T* is the radiocarbon ages of the time slices. The metric indicates the number of points where the model extent does not match the reconstruction for any of the time steps *t* within the uncertainty range for the time slice *T* (*Tmin*, *Tmax*).


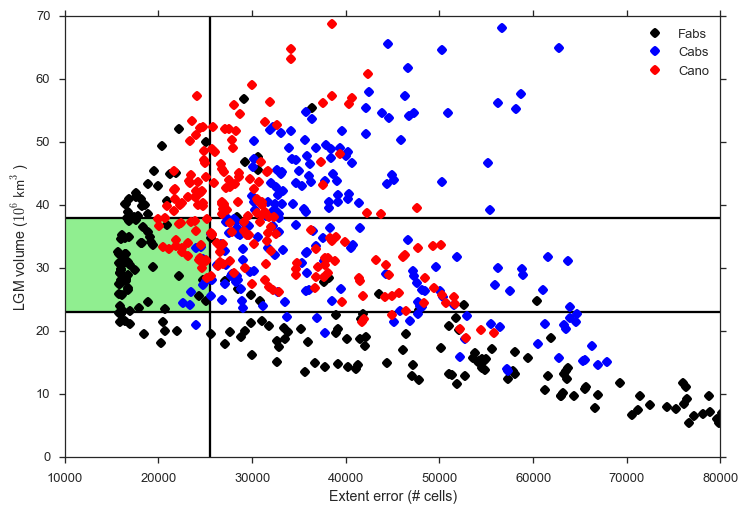


**Figure S1:** Last Glacial Maximum (LGM) ice volume versus ice sheet extent for all simulations showing the Fabs (black; FAMOUS F-21k absolute forcing), Cabs (blue; CCSM3 T-21k absolute forcing), and Cano (red ; CCSM3 T-21k anomaly forcing) ensemble members that fall within the selection criteria (green box).


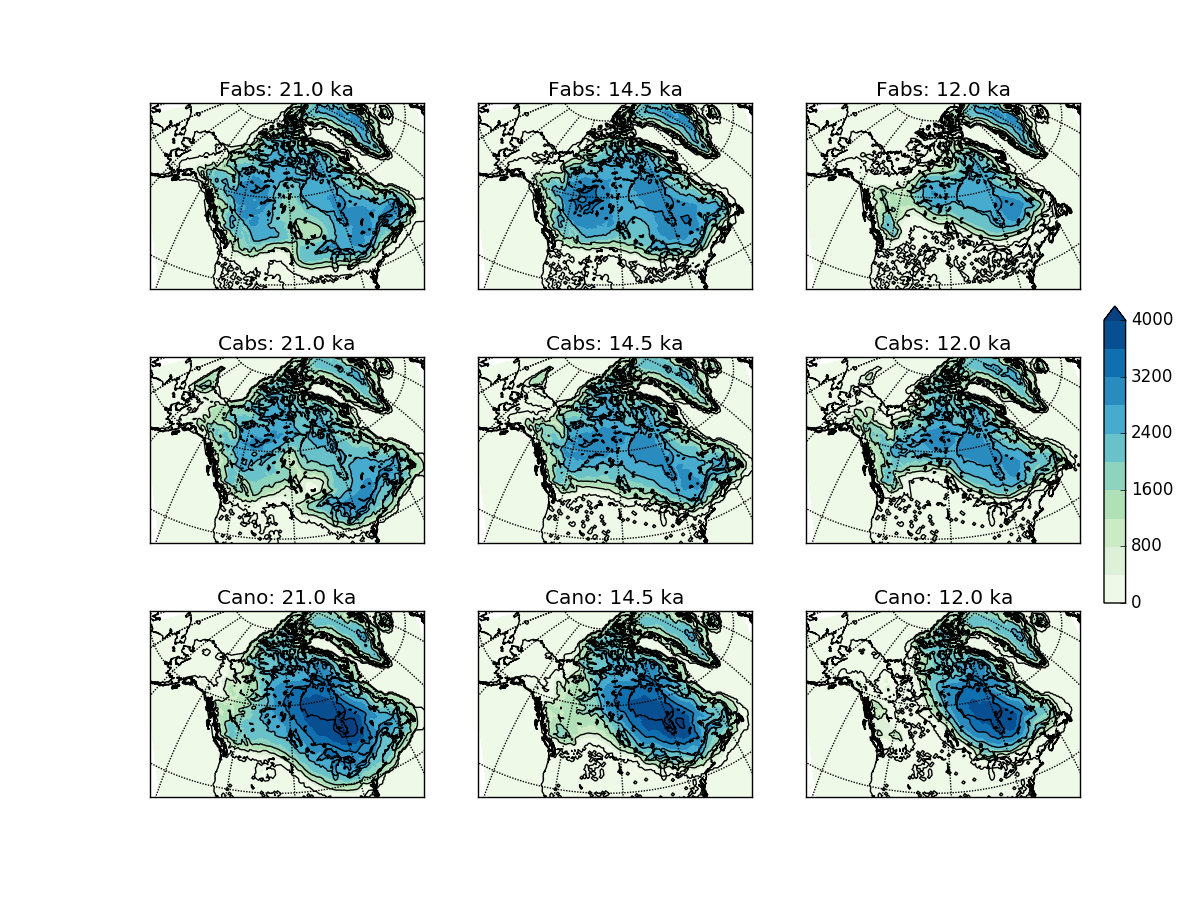


Figure S2: Ice thickness (m) averaged over the Not Ruled Out Yet ensemble members for the Fabs (FAMOUS absolute), Cabs (CCSM3 absolute) and Cano (CCSM3 anomaly) ensembles at 21, 14.5 and 12 ka.

| **Parameter** | **Min** | **Max** | **unit** |
| --- | --- | --- | --- |
| Flow factor | 1 | 10 | - |
| Geothermal Heat flux | 0.020 | 0.090 | W m^-2^ |
| Basal sliding parameter | 0.5 | 20 | mm yr^-1^ Pa^-1^ |
| Mantle relaxation time | 300 | 9000 | Year |
| Positive degree day factor for snow | 0.002 | 0.006 | m d^-1^ K^-1^ |
| Positive degree day factor for ice | 0.007 | 0.020 | m d^-1^ K^-1^ |
| Lapse rate | 4 | 8.2 | K km^-1^ |

Table S1. Range of values of uniform distributions used to sample ice sheet model parameters.
